# Brazilian adaptation of the Hotel Task: A tool for the ecological assessment of executive functions

**DOI:** 10.1590/1980-57642015DN92000010

**Published:** 2015

**Authors:** Caroline de Oliveira Cardoso, Nicolle Zimmermann, Camila Borges Paraná, Gigiane Gindri, Ana Paula Almeida de Pereira, Rochele Paz Fonseca

**Affiliations:** 1 Department of Psychology, Pontifícia Universidade Católica do Rio Grande do Sul, Porto Alegre RS, Brazil.; 2 Department of Psychology, Universidade Federal do Paraná, Curitiba PR, Brazil.

**Keywords:** neuropsychology, executive functions, adaptation, Hotel Task

## Abstract

Over recent years, neuropsychological research has been increasingly concerned with the need to develop more ecologically valid instruments for the assessment of executive functions. The Hotel Task is one of the most widely used ecological measures of executive functioning, and provides an assessment of planning, organization, self-monitoring and cognitive flexibility.

**Objective:**

The goal of this study was to adapt the Hotel Task for use in the Brazilian population.

**Methods:**

The sample comprised 27 participants (three translators, six expert judges, seven healthy adults, ten patients with traumatic brain injuries and one hotel manager). The adaptation process consisted of five steps, which were repeated until a satisfactory version of the task was produced. The steps were as follows:

(1) Translation;
(2) Development of new stimuli and brainstorming among the authors;
(3) Analysis by expert judges;
(4) Pilot studies;
(5) Assessment by an expert in business administration and hotel management.

**Results:**

The adapted version proved adequate and valid for the assessment of executive functions. However, further research must be conducted to obtain evidence of the reliability, as well as the construct and criterion validity, sensitivity and specificity, of the Hotel Task.

**Conclusion:**

Many neurological and/or psychiatric populations may benefit from the adapted task, since it may make significant contributions to the assessment of dysexecutive syndromes and their impact on patient functioning.

## Abstract (pt)

A busca por validade ecológica dos instrumentos neuropsicológicos vem recebendo uma atenção especial nos últimos anos, especialmente daquelas ferramentas que avaliam funções executivas. A Tarefa do Hotel é uma das tarefas ecológicas de exame executivo mais reconhecida internacionalmente, por examinar planejamento, organização, automonitoramento e flexibilidade cognitiva.

**Objetivo:**

O objetivo deste estudo é apresentar o processo de adaptação da Tarefa do Hotel para uma versão brasileira.

**Método:**

A amostra constitui-se de 27 indivíduos (três tradutores, seis juízes especialistas, sete adultos saudáveis, dez pacientes pós traumatismocranioencefálico e um gerente de hotel). Cinco etapas foram conduzidas, replicadas ao longo do processo:

(1) tradução,
(2) desenvolvimento de novos estímulos e *brainstorming* entre os autores,
(3) análise de juízes especialistas em neuropsicologia,
(4) estudos pilotos e
(5) julgamento de expert em administração e hotelaria.

**Resultado:**

A versão adaptada mostrou-se adequada e válida para avaliar componentes executivos, sendo necessários estudos futuros em busca de evidências de fidedignidade, validades de construto e de critério, sensibilidade e especificidade, considerando-se as particularidades de uma ferramenta ecológica.

**Conclusão:**

Muitas populações clínicas neurológicas e/ou psiquiátricas poderão se beneficiar do exame com a Tarefa do Hotel, por esta auxiliar no diagnóstico de síndrome disexecutiva relacionada à demanda de componentes executivos no cotidiano.

## INTRODUCTION

Over recent years, considerable research and clinical attention has been dedicated to the associations and dissociations between executive functions (EF). This term refers to the ability to engage in goal-oriented, voluntary and independent behaviors.^1^ However, researchers have not yet agreed on a theoretical model of EF, nor is there a consensus on which specific components comprise EF. One of the greatest challenges posed by the absence of sufficiently consolidated models of EF is the difficulty in operationalizing assessment procedures and rehabilitation programs for executive functioning. Although several instruments have been designed in an attempt to provide an accurate assessment of EF, given the complexity of these abilities and the extent of the controversy as to their nature, even traditional assessment instruments have been criticized for their lack of specificity. Although they tend to be sensitive for detecting executive impairments, they are not always accurate in predicting and evaluating the impact of these impairments on daily functioning.^2-4^ As a result, researchers and clinical practitioners have been increasingly concerned with the need to develop more ecologically valid instruments for the assessment of EF.^5-7^

The term "ecological validity" refers to the functional and predictive relationship between performance on neuropsychological assessments and impairments in daily living tasks.^8,9^ Neuropsychological research has been increasingly concerned with the development and adaptation of ecologically valid measures of EF. Some of these instruments include the Behavioral Assessment of the Dysexecutive Syndrome (BADS),^10^ the Multiple Errands Task,^11^ the Iowa Gambling Task^12^ - adapted to Brazilian Portuguese^13,14^ and the Hotel Task.^15^

The Hotel Task focuses on the assessment of the following executive components: planning, organization, self-monitoring and cognitive flexibility.^15^ The task simulates a real-life situation which requires the involvement of multiple executive resources for its correct resolution. It is an ecologically valid and standardized task which has proved to be sensitive in the assessment of several clinical conditions such as frontotemporal dementia,^16^ multiple sclerosis,^17^ attention deficit/hyperactivity disorder,^18^ and bipolar disorder.^18^

There is also a pronounced lack of ecologically valid measures of EF adapted and 'normed' for use in Brazil. Therefore, to address this gap in assessment tools, the present study aimed to adapt the Hotel Task^15^ for use in Brazilian Portuguese, ensuring its adequacy for the social, cultural, linguistic and cognitive characteristics of the target population.

## METHOD

The adaptation process involved the following stages:

[1] Translation;
[2] Development of new stimuli and brainstorming among the authors;
[3] Analysis by expert judges;
[4] Pilot studies;
[5] Assessment by an expert in business administration and hotel management. Each stage was performed as many times as required to ensure the quality of the final product of the adaptation process.

**Participants.** The sample comprised 27 participants, consisting of three translators, six expert judges, 17 participants in the pilot study, and one hotel manager, whose contributions to the study will be explained in the Procedures section. Table 1 presents the characteristics of each sample.

**Table 1:** Sample characteristics by adaptation phase of the Hotel Task.

Phase number	Phase name	Participants	Descriptive data	Task version
1	Direct translation	02 translators	Bilingual neuropsychologists with background in EF* neuropsychology and proficiency in English and native Brazilian Portuguese	
	Compared translation	01 expert translator for consensus	Neuropsychologist and speech therapist with proficiency in English and native Brazilian Portuguese	
2	Author brainstorm - phase 1	06 - Discussion of semantic adjustments to instructions and task stimuli	05 Neuropsychologists and 01 speech therapist proficient in neuropsychology and instrument adaptation	VERSION 1
3	Expert judge analysis - phase 1	04 expert judges - Evaluation of instructions and task stimuli adequacy	03 Neuropsychologists and 01 Speech therapist	
4	Author brainstorm - phase 2	06 - Discussion of adjustments to instructions and task stimuli after suggestions by expert judges	05 Neuropsychologists and 01 speech therapist proficient in neuropsychology and instrument adaptation	VERSION 2
5	Pilot study 1	01 healthy adult	Age (years): 20 Education (years): 13 Sex: Female	
6	Author brainstorm - phase 3	06 - Discussion of adjustments to instructions, task stimuli and scoring after observations in Pilot study 1	05 Neuropsychologists and 01 speech therapist proficient in neuropsychology and instrument adaptation	VERSION 3
7	Expert judge analysis - phase 2	02 expert judges	02 Neuropsychologists	
	Pilot study 2	05 healthy adults	Age in years, mean: 25.02 Education in years, mean: 16.2 Sex: 03 Male and 02 Female	
8	Author brainstorm - phase 4	06 - Discussion of adjustments to instructions, task stimuli and scoring	05 Neuropsychologists and 01 speech therapist proficient in neuropsychology and instrument adaptation	VERSION 4
9	Pilot study 3	01 healthy adult	Age in years: 54 Education in years: 18 Sex: Female	
10	Author brainstorm - phase 5	06 - Discussion of adjustments to instructions, task stimuli and scoring	05 Neuropsychologists and 01 speech therapist proficient in neuropsychology and instrument adaptation	VERSION 5
11	Appraisal of expert judge analysis	01 Professional expert	01 Expert in business administration with emphasis in hotel management and 11 years of experience as hotel manager	
12	Pilot study 4	10 TBI** patients	Age in years, mean (SD#): 37.09 (10.29) Education in years, mean (SD#): 9.36 (5.73) Sex: 08 male and 02 Female	

* Executive function;

** Traumatic brain injury;

# Standard deviation.

Control subjects were recruited using convenience sampling within university and community-based settings. A group of patients with TBI was also recruited from the patient records of public hospitals in the cities of Porto Alegre and Curitiba.

**Procedures and instruments.** In accordance with ethical guidelines for human research, participation was voluntary and all participants provided written informed consent. The present study was also approved by the Research Ethics Committee of the Pontifical Catholic University of Rio Grande do Sul. Participants were assessed in a single session lasting approximately one hour, during which the Hotel Task, as well as sample characterization measures and screening instruments, were administered. Patient characteristics were assessed using a sociodemographic and cultural questionnaire.^19^ The goal of this procedure was to test the instrument in a real data collection scenario, so as to estimate its duration, and verify the comprehensibility of its instructions and stimuli. Both the adapted and original versions of the Hotel Task comprise five activities involved in hotel management. The materials required to perform the task were laid out on a table in front of the subjects, who were provided the following instructions:

"In this task you are asked to imagine that you are working in a hotel. Your manager would like to see how you perform each of these five activities in the next 15 minutes as part of the hiring process for working at the hotel. Your main goal is to do at least some of these five tasks over the next 15 minutes. Each of the tasks may well take longer than 15 minutes alone to complete. Therefore, it is impossible to complete all the tasks within the time limit. The most important thing is to try and do something from each task within the available time period, while keeping track of the time with this clock. Did you understand the instructions? I will now explain each of the tasks. Please pay close attention!"

The following tasks were included in the instrument:

*Compiling individual bills task* – Participants are asked to post guest charges on bills, writing down the total amount of money spent by each individual as well as the value of each service or product purchased.

*Sorting the charity collection task* – The goal of this task is to separate foreign currency coins from Brazilian Real (R$) coins.

*Looking for promotions in menu task* – Participant was given a menu from the hotel restaurant, and a list of the 20 promotions of the month. They were asked to locate each of these dishes or drinks in the menu, and write down their prices in the promotion list.

*Sorting conference labels task* – Participant was asked to organize 80 nametags into alphabetical order.

*Proof reading the hotel brochure* – Participants were asked to identify and rectify errors (words with double letters) in the new hotel brochure.

In addition to these tasks, the participant was asked to make two wake-up calls to hotel guests by pressing two buttons on a telephone at two different time points.

**Instruments and procedures per adaptation step.** Each version of the task produced throughout the adaptation procedure was analyzed by the authors. The details of each version of the task are described in Table 2.

**Table 2:** Phases for Hotel Task adaptation.

Phases	Specification
1 - Translation	Instructions and stimuli were translated and adapted to the Brazilian social and cultural context. The overall procedure was divided into two sub-steps: a) direct translation by two English-proficient translators; and b) translation comparison by an expert in the assessed constructs and knowledge in the languages involved.
2 - Development of new stimuli and author brainstorm - phase 1	Authors developed stimuli and performed semantic adjustments to instructions from the original version (Manly et al., 2002) and the study of the applicability of the Hotel Task by Torralva et al. (2012). The first version of the Hotel Task was developed (Version 1).
3 - Expert judge analysis - phase 1	Professionals analyzed the adapted stimuli from the previous step and instructions for each task. At first, each judge was required to individually verify whether each stimulus was appropriate for the respective instruction, suggesting changes where necessary.
4 - Development of new stimuli and author brainstorm - phase 2	Based on expert analysis and suggestions, the authors modified several stimuli and a new version was developed (Version 2).
5 - Pilot study	The first pilot study was conducted with the second version of the task in order to test the instrument in a real assessment situation, as well as to analyze potential faults and stimuli adequacy. A healthy young adult participated.
6 and 7 - Development of new stimuli and author brainstorm - phase 3; and Expert judge analysis - phase 2 and Pilot study 2	The authors made further adjustments after observations from the first pilot study (Version 3). Furthermore, a second expert judge analysis was concurrently conducted via a pilot study with Version 3 and five healthy adults.
8 and 9 - Development of new stimuli and author brainstorm - phase 4; and Pilot study 3	The authors made further adjustments after suggestions by the expert judges and observations from the pilot study (Version 4). The fourth version of the instrument was utilized with a healthy adult in phase 9.
10 and 11 - Development of new stimuli and author brainstorm - phase 5; and assessment by expert judge analysis	Further adjustments were made after observations and analysis of the third pilot study (Version 5). A professional in business administration with emphasis in hotel management analyzed the proposed activities. Each task was explained and the judge evaluated whether instructions and stimuli were consistent with the reality of Brazilian hotels.
12 - Pilot study 4	The fifth version was utilized with 10 patients with traumatic brain injury (TBI). This population was selected for having a high prevalence of executive dysfunction.

**Data analysis.** All stages of the adaptation process were descriptively analyzed, and any changes made to the task were carefully described. The agreement between judges was descriptively assessed using the methods described by Fagundes^20^ and Andres and Marzo.^21^

## RESULTS

Each stage in the process of adapting the Hotel Task to Brazilian Portuguese is described below. The versions of the task produced after each phase are presented in Table 3.

**Table 3:** Versions of the Hotel Task through adaptation phases.

Version	Compiling individual bills task	Sorting the charity collection task	Looking for promotions in menu task	Sorting conference labels task	Proof reading the hotel brochure	Waking up guests	Instructions	Scoring
VERSION 1	Development of two billing lists: one with the costs of services provided to all guests and another for individual guest billing	Selection of 50 coins of Brazilian currency and 50 coins of foreign currency	Development of a list of local companies selected from a public telephone list. The participant was required to search the public list for companies’ telephone numbers	Development of 100 name tags with the name and surname from participants of a meeting	A hotel folder was developed. It comprised 509 words, 17 orthographic errors, font size 12, in color	A box was used as the garage remote control	A general instruction and further instructions for each task	Variables: Number of tasks started; task time; Number of times garage button was pressed; Time deviations in opening and closing garage doors; Correct responses versus number of tries on each task
VERSION 2	An example was added to task instructions	No modification	Task was maintained, however each Brazilian state were required to create its own list of companies	No modification	No modification	No modification	Instructions were all modified; in the adapted version, instructions were only read aloud to the patient, with no written version available for further consultation	Qualitative analyses were included
VERSION 3	No modification	Number of coins was modified to 45 each. Besides sorting foreign from Brazilian coins, participants had to sort Brazilian coins according to their value into predefined spaces	No modification	No modification	No modification	No modification	No modification	No modification
VERSION 4	We increased font size	Number of coins was altered to 100 Brazilian coins and 10% target coins (foreign coins)	We modified task stimuli. A restaurant menu with promotional meals was developed. Task objective was to find the promotional meals	We opted to insert the first name followed by the surname. Number of tags was reduced to 80 and font size was increased.	Font size was increased and writing was printed in black font. The amount of errors corresponded to around 10% the number of words (539 words and 62 errors)	Instead of using a box as remote control, participants were required to press two telephone buttons in order to wake up guests.	Participants also received a written version to follow during reading of instructions. For each task, an instruction chart was available	No modification
VERSION 5	Same version was maintained, with an increase in font size to 16	Number of coins was increased to 198 Brazilian and 22 foreign coins. Number of coins by value and nationality was stimulated	The Promotional meals task was officially established	Version 4 was maintained	Version 4 was maintained	The idea of waking up guests was maintained	No modification	No modification

**Phases 1.** The task instructions and stimuli were independently translated from English to Brazilian Portuguese by two translators. Differences between the translations were resolved by a third professional, who adapted the wording of the task for the social and linguistic characteristics of the Brazilian population.

**Phases 2.** Stimuli were created and adapted based on participant familiarity, and with the aim of making the task ecologically valid and as close as possible to the reality of a hotel. The number of coins in the sorting task was reduced, as was the number of pages and typing errors in the brochure used in the proofreading task (in the original Hotel Task, the brochure had nine pages containing 130 errors, while the adapted brochure was three pages long and contained 17 errors, maintaining the page-to-error ratio of the original instrument). The version of the Hotel Task produced after this stage of the adaptation process is described in Table 3.

**Phases 3.** The tasks were descriptively analyzed by the expert judges, who agreed as to the adequacy of the activities and the executive abilities assessed by each. However, changes were made to the task instructions, its scoring system and to the Compiling Customer Bills and Telephone Book tasks, as shown in Table 3.

**Phases 4 and 5.** The authors revised and modified the task based on the analysis and suggestions made by the expert judges, and the following two changes were made: inclusion of an example in the Compiling Customer Bills task and creation of a separate telephone book for each location in which the instrument was used. The instructions for the tasks were also made clearer and more precise. These changes resulted in the production of a second version of the adapted Hotel Task (Table 3), which was used in a pilot study. The participant was able to perform all tasks and remembered to press the garage door buttons as requested. The pilot study revealed that the number of coins in the sorting task was too small, such that the task could be completed in a very short period of time. The participant also had trouble handling the telephone book, which led him to spending longer on this task than expected. After completing the Hotel Task, the participant mentioned his unfamiliarity with telephone books, to which he attributed his difficulties in the aforementioned task.

**Phases 6 and 7.** After the first pilot study, some changes were made to the task stimuli and instructions. The number of coins in the sorting task was increased, and after two expert judges had reevaluated the task, a second pilot study was conducted. The judges suggested that the telephone book task be replaced so as to assure the ecological validity and practicality of the Hotel Task. For the same reason, the garage door task was replaced by the two wake-up calls, which the participant was instructed to make by pressing two buttons on a telephone. In the second pilot study, most healthy subjects were able to perform all five tasks, and remembered to call the two hotel guests. However, they still had difficulty handling the telephone book. The fact that, in the conference label task, participants were asked to alphabetically order the nametags by last name also led to some problems in this activity. Furthermore, the font size in some of the tasks interfered with participant performance, as did the length of the instructions.

**Phases 8.** Based on the suggestions made by the expert judges and on the analysis of the results obtained in the second pilot study, further modifications were made to some of the tasks in the instrument. The telephone book task was replaced by the "Looking for monthly promotions in the hotel menu" task, whose underlying procedure was similar to that of the telephone book task. In this activity, the participant was given a list of the monthly promotions in the hotel restaurant, and was asked to look up each dish or drink on a menu and write down its price. In the conference label task, participants were also instructed to order the nametags alphabetically by first rather than last name, a more common procedure in Brazil. The sizes of the labels were also changed to ensure that participants had no trouble handling or reading the cards. The coin and brochure tasks were changed so that the target-to-distractor ratio was 1:10, as is commonly adopted in canceling tasks. Due to the complexity and length of the Hotel Task instructions, a written copy of the directions was provided to participants so they could follow these while the researcher read them out prior to the administration of the task, as in its original English version. Additionally, a card with the instructions for each task was laid out next to its corresponding materials, to be consulted by participants whenever necessary.

**Phases 9 and 10.** The participant who was administered the Hotel Task in the third pilot study displayed satisfactory performance, and was able to distribute his time among the five tasks. The coin task was found to be too easy for participants, who were able to complete it within three minutes. Therefore, the only change made to the task in stage 10 of the adaptation process was an increase in the number of coins to 198 Real coins and 22 foreign coins, as described in Table 5.

**Table 5:** Description of the original Hotel Task and of the final Brazilian adapted version.

Version by Manly et al. (2002)	Adapted version
Compiling individual bills. Two types of material are presented: The first represents the till roll from the hotel register listing services provided to the guests and their costs. The second are individual bill forms for each of the guests. The till roll needed to be scanned for services used by a particular guest and then these items transferred to individual bills. Eight guest bills each featuring 10 separate items would need to be compiled.	Compiling customer bills. The task was identical to the original version, except for the inclusion of an example before starting the task.
Sorting the charity collection. One box containing 196 coins, of which 21 are of foreign origin. The goal in this task is to sort the British from the foreign coins. The British coins comprised: 5×1p, 4×2p, 96×5p, 46×l0p and 24×20p.	Sorting the charity collection task. A total of 198 Brazilian coins and 22 of foreign currency. The goal is to sort foreign coins from Brazilian coins. Brazilian coins available are: 7x1.00, 16x0.50, 45x0.25, 65x0.10 and 65x0.05.
Looking up telephone numbers. Participants are provided with a list of 34 local companies and asked to find and note down their telephone numbers using the regional Yellow Pages phone directory.	Looking for promotions in menu task. A list of 20 monthly promotions is provided and the participant is asked to search for matching prices in the menu.
Sorting conference labels. Participants are provided with a pile of 100 labels, each with the name of a guest attending a conference. Prior to each administration, the pile is shuffled and participants are asked to sort the cards into alphabetical order based on the surname of each guest.	Sorting conference labels task. Eighty labels are provided and should be arranged in alphabetical order according to the first name of the respective guest.
Proof Reading the hotel leaflet. A leaflet of nine pages with specific typos repetition of letters. A total of 130 (4%) is presented. Participants are asked to mark the errors with a pen.	Proof reading the hotel brochure. A two-page brochure with specific typos repetition of letters. A total of 62 errors (10%) is presented. Participants are asked to identify and rectify errors.
Opening and closing the garage doors. At two pre-defined times (6 and 12 min after beginning the task), the participants are asked to remember to open and close the hotel garage doors, in order to allow deliveries. The door is opened by pressing a red button and closed by pressing a black button, both mounted on a single button box placed on the desk.	Waking up guests. Participants are asked to perform two wake-up calls to hotel guests by pressing two buttons on a telephone at two different time points (6 and 12 min after starting the task).

**Phases 11.** A hotel manager assessed the similarity of the stimuli and instructions of the Hotel Task with those in his workplace, and reported a similarity of 100% between the task and the objects found in a real-world hotel setting.

**Phases 12.** This was the last stage in the adaptation process, since the version of the Hotel Task produced after stage 11 was considered by the authors to be appropriate both for healthy individuals and for patients with neurological conditions. Table 4 shows the performance of patients who took part in the last pilot study. Table 5 describes the original version of the Hotel Task and the latest adapted version.

**Table 4:** Participant performance in the Pilot study with the Hotel Task.

Task	Performance of TBI* patients M** (SD^#^)
Number of tasks performed	3.10 (1.66)
Total time in the Compiling customer bills task, in seconds	143.90 (207.40)
Total correct responses in the Compiling customer bills task	7.00 (12.42)
Total number of attempts in the Compiling customer bills task	7.20 (13.00)
Total time in the Sorting the charity collection task, in seconds	210.10 (187.37)
Total correct responses in the Sorting the charity collection task	122.80 (187.39)
Total number of attempts in the Sorting the charity collection task	126.90 (102.57)
Total time in the Proof reading the hotel brochure, in seconds	51.50 (78.74)
Total correct responses in the Proof reading the hotel brochure	6.30 (9.44)
Total number of omissions in the Proof reading the hotel brochure	6.80 (14.63)
Total time in the Sorting conference labels task, in seconds	368.30 (244.73)
Total correct responses in the Sorting conference labels task	22.50 (22.69)
Total number of attempts in the Sorting conference labels task	34.20 (29.74)
Total time in the Looking for promotions in menu task, in seconds	71.30 (101.85)
Total correct responses in the Looking for promotions in menu task	2.00 (4.13)
Total number of attempts in the Looking for promotions in menu task	2.20 (4.13)
Time for waking up the first guest, in seconds	205.20 (245.70)
Time for waking up the second guest, in seconds	365.80 (387.44)

* Traumatic Brain Injury;

** Mean;

# Standard deviation;

The scores of patients with TBI varied widely, as shown in Table 4. On average, patients engaged in three out of the five tasks available. There were no floor or ceiling effects. Healthy participants were administered a similar version of the task, and were also able to perform all five tasks within the 15 minutes provided. A comparison between the first and final versions of the task is given in Table 5.

## DISCUSSION

The present study described the adaptation of the Hotel Task for use in the Brazilian population. This was the first stage in the instrument's validation process, and an extremely important source of evidence of the adequacy of this instrument for use in research and clinical practice in Brazil.

There is no consensus in the literature as to the ideal method to be used in the cross-cultural adaptation of neuropsychological assessment instruments. However, Fonseca et al.^22^ found that, in the case of non-verbal measures, the adaptation process consisted mostly of the translation and back-translation of instructions of the original task.^14,23,24^ Fonseca et al.^22^ suggested that these procedures be followed by analysis by expert and non-expert judges as well as pilot studies. After each stage of this process, the authors of the instrument should analyze the task and make any necessary adjustments. All of these phases were followed in the present study, as the hotel manager, who had no professional experience with neuropsychology, contributed to the process as a non-expert judge. Since there are no clear descriptions of the Hotel Task in the literature, the focus of the adaptation process was the pilot study rather than the analysis by expert and non-expert judges.

A similar phenomenon occurred in the adaptation of the Iowa Gambling Task for use in Southern Brazil: in this case, the task instructions were translated by an English-speaking professional before being evaluated by two judges with a background in neuropsychology and knowledge of the English language, who ensured the sociocultural adequacy of the stimuli; the last step in the adaptation process was a pilot study of the adapted task.^13^

In the present study, two translators worked independently on the instructions of the *Hotel Task.*^15^ However, translation alone may not be able to guarantee the equivalence between an adapted task and its original version,^25,26^ and therefore may not always lead to successful cross-cultural adaptations of assessment instruments.^22^ Acevedo et al.^27^ suggested that adapted translations may be more adequate than literal ones, where the latter do not involve the adjustment of terms to the local culture, compromising the comprehensibility of task stimuli and instructions. Adapted translations involve modifications to the terminology and semantic structure of instructions and stimuli to ensure their comprehensibility and familiarity.

Analyses by expert judges are a commonly used method for the cross-cultural adaptation of assessment instruments. Such procedures were involved, for instance, in the adaptation of the Montreal Communication Assessment Battery,^26^ the Brief Montreal Communication Assessment Battery – MAC-B,^25^ and of the WAIS-III and WISC-III^28^ for use in Brazil. The judges may suggest changes to the task which can be evaluated and implemented by its authors. Due to the complexity of the task used in the present study, expert judges had to be consulted more than once in the adaptation process and, as previously mentioned, the pilot investigation was crucial for assessing the need for modifications to the task. A total of six pilot studies were conducted in the present investigation. According to Fonseca et al.,^22^ the neuropsychological literature does not specify an ideal sample size for pilot studies. In the present study, healthy individuals with different ages and educational levels as well as patients with TBI were recruited for the pilot studies.

The small number of participants in the pilot study may constitute a limitation of the present study. However, as there is no consensus in the literature as to the ideal sample size for pilot studies of adapted neuropsychological instruments, we had no way of assessing the adequacy of our sample size. Additionally, the length and complexity of Hotel Task instructions may have influenced the performance of patients with low educational levels or severe neurological conditions on the task. Impairment in EF associated with these features may impair comprehension of task instructions as well as performance on the Hotel Task, since the activities involved in the instrument rely on the use of multiple EF for their successful completion.

In spite of these limitations, the adaptation of the Hotel Task for use in the Brazilian population was successful, and all recommended stages for the cross-cultural adaptation of neuropsychological instruments were closely followed. We suggest that further research be conducted to assess the psychometric properties and applicability of this instrument in other clinical populations, as well as its ability to differentiate between clinical conditions such as dementias. The Hotel Task may be especially sensitive to patients with frontotemporal dementia, for instance, since executive impairments are a hallmark of this disorder.^29^ Furthermore, we also suggest that a more concise and less complex version of the Hotel Task be developed, so that it can be used in patients with lower educational levels and more severe neurological conditions. Further research should also look more deeply into the development of shorter ecologically valid tasks which could, for instance, be used in bedridden patients.
